# On the Application of Time Frequency Convolutional Neural Networks to Road Anomalies’ Identification with Accelerometers and Gyroscopes

**DOI:** 10.3390/s20226425

**Published:** 2020-11-10

**Authors:** Gianmarco Baldini, Raimondo Giuliani, Filip Geib

**Affiliations:** 1 European Commission, Joint Research Centre, 21027 Ispra, Italy; raimondo.giuliani@ec.europa.eu; 2 Faculty of Electrical Engineering, Czech Technical University in Prague, 160 00 Prague, Czech Republic; filip9geib@gmail.com

**Keywords:** convolutional neural networks, deep learning, time-frequency, Inertial Measurement Unit (IMU), road anomalies

## Abstract

The detection and identification of road anomalies and obstacles in the road infrastructure has been investigated by the research community using different types of sensors. This paper evaluates the detection and identification of road anomalies/obstacles using the data collected from the Inertial Measurement Unit (IMU) installed in a vehicle and in particular from the data generated by the accelerometers’ and gyroscopes’ components. Inspired by the successes of the application of deep learning to various identification problems, this paper investigates the application of Convolutional Neural Network (CNN) to this specific problem. In particular, we propose a novel approach in this context where the time-frequency representation (i.e., spectrogram) is used as an input to the CNN rather than the original time domain data. This approach is evaluated on an experimental dataset collected using 12 different vehicles driving for more than 40 km of road. The results show that the proposed approach outperforms significantly and across different sampling rates both the application of CNN to the original time domain representation and the application of shallow machine learning algorithms. The approach achieves an identification accuracy of 97.2%. The results presented in this paper are based on an extensive optimization both of the CNN algorithm and the spectrogram implementation in terms of window size, type of window, and overlapping ratio. The accurate detection of road anomalies/obstacles could be useful to road infrastructure managers to monitor the quality of the road surface and to improve the accurate positioning of autonomous vehicles because road anomalies/obstacles could be used as landmarks.

## 1. Introduction

Detection of road anomalies like potholes, road cracks, and road safety features/obstacles like speed bumps (also called speed humps) has been investigated by the research community in recent years using a variety of sensors including cameras, Light Detection And Ranging (LiDAR)s, and Inertial Measurement Units (IMU)s. One of the main applications for the detection of road anomalies is the monitoring of the road conditions, which can be used to repair the road surface once a road anomaly (e.g., pothole) is detected or to improve the comfort and safety of the vehicle in Advanced Driver Assistance Systems (ADAS). An additional aspect is to use the identification of road anomalies as landmarks to enhance maps and improve localization to a high degree of accuracy. The upcoming evolution of modern vehicles to autonomous vehicles could benefit from this information and use it for a variety of purposes. It could be used to improve the position of the autonomous vehicle. It could be used improve its travel plan for the comfort of the passengers (e.g., the autonomous vehicle could slow down before a known pothole). It could even be used to mitigate cybersecurity attacks on the positioning information (e.g., the position information would be correlated with the detected road anomaly to mitigate Global Navigation Satellite Systems (GNSS) spoofing attacks). The use of the position of the road anomaly to support localization algorithms was already mentioned in [1,2]. In addition, studies like [3] suggest that artificial position measurements obtained by detecting road anomalies associated with map locations will be increasingly used in the automotive sector and automated vehicles also in synergy with Simultaneous Localization And Mapping (SLAM) techniques by the robotics community.

Detection of road anomalies can be performed using different techniques including machine learning algorithms [4,5], and in recent times, Deep Learning (DL) algorithms have been adopted with significant success. In many cases, DL is used to detect and identify road anomalies on the basis of the images collected by the camera installed on the vehicle [6,7]. Processing of data images can be a time consuming and error prone task [5], and it is limited by the lighting (e.g., darkness) or environmental conditions (e.g., fog, rain) [8]. An alternative approach, which is not affected by these issues, is to use the data provided by the IMU, and DL was applied to such data in recent studies [9]. Then, the application of DL for road anomaly detection using data from accelerometers and gyroscopes is a recent research area, which is gaining significant momentum in the research community, and it is further explored in this paper.

This paper proposes the combination of time-frequency transform and CNN (called CNN-SP) for the detection and identification of road anomalies in the road infrastructure, which has not been proposed in the literature yet (to the knowledge of the authors) for this specific problem. As shown in the results provided in this paper, CNN-SP provides a superior classification performance to the direct application of CNN to the original time domain signal. This paper uses a relatively large set of vehicles (12) in the data collection phase to improve generalization of the results. The application of CNN-SP is evaluated using an experimental dataset collected by the authors with many hours of driving on a realistic road path with various types of road anomalies and obstacles. The approach is evaluated using data both from the accelerometer and the gyroscope. Finally, this study provides an extensive evaluation of the different sampling rates on the identification accuracy.

The structure of this paper is as follows: Section 2 provides a literature review on the detection of road anomalies using sensors installed on the vehicles with a particular focus on accelerometers and gyroscopes. Section 3 describes the materials and methods used for the analysis including the description of the adopted machine learning algorithms and the related evaluation metrics. Section 4 provides the results of the analysis including the optimization of the CNN-SP approach and a comparison among the results provided by the different machine learning algorithms. Finally, Section 5 provides the conclusions.

## 2. Literature Review

Detection of road anomalies and road surface conditions using the sensors installed on the vehicle has been investigated by the research community in recent years. Detection of road anomalies through cameras can provide high accuracy especially with the recent application of deep learning. Examples of the application of deep learning (and convolutional neural networks in particular) in combination with camera images to assess road surface condition were provided in [6,7]. The use of data images can be a time consuming and error prone task [5], and it is limited by the lighting (e.g., darkness) or environmental conditions (e.g., fog, rain) [8]. For this reason, this paper does not use an approach based on camera images, and other sensors are used.

The application of accelerometers, gyroscopes, and magnetometers to detect road anomalies like potholes and obstacles (e.g., speed humps) has been investigated by the research community in recent years [10]. This is also due to the decreasing costs of IMUs and their increasing use in the automotive sector either because they are inserted and used in the vehicle systems or because smartphones (which are equipped with IMUs) can be deployed and installed in vehicles. A very recent and detailed survey on the use of accelerometers and gyroscopes (as inertial sensing sources of information) was presented in [4] where an analysis focused on identifying methods that capture signals provided by inertial sensors such as accelerometers and gyroscopes to recognize transient or persistent events associated with the vehicle’s movement was presented. The results of the survey show that a limited amount of the reviewed papers used time-frequency analysis, and no paper in the review used the combination of the time-frequency and convolutional neural networks, as proposed in this paper.

Detection of road anomalies like potholes using accelerometers was implemented by the authors in [11,12] where a high detection rate was achieved using the data collected by the accelerometers in the Z direction, but no deep learning approach was used. In a similar way, the authors in [13] used a generic smartphone application reading data from built-in accelerometers sensors to map and measure the locations of potholes and speed bumps, which can be used to evaluate the road conditions. The data were collected in a cloud system, where the analysis was performed. The authors in [5] proposed the Android application RoadSense, which automatically predicts the quality of the road based on a triaxial accelerometer and a gyroscope. The study used frequency domain features for the classification combined with different machine learning algorithms. As in previous papers, the focus was on pothole detection to monitor the smoothness of the road surface, but the identification of the road anomalies was not attempted.

Four recent works are very similar to the study presented in this paper. In [14], different road anomalies like bump, pothole, and normal conditions (flat road) were detected and identified using an improved Gaussian background model and the K Nearest Neighbor (KNN) algorithm applied to the accelerometers’ recording. The described approach provides a high accuracy (96.03%) of recognition of the road surface pothole and a high accuracy of the road surface bump of 94.12%. In comparison to [14], this paper applies a more sophisticated deep learning approach in combination with a time-frequency transform, which is shown to provide a higher identification accuracy than the time domain representation (i.e., original accelerometer signal). In addition, gyroscope data were also used in addition to the accelerometer data. Finally, a larger set of 12 vehicles is used than the two vehicles used in [14], which improves the generalizations of the results.

The authors of [8] proposed a novel approach to identify the profile of a pothole, which is a more challenging task than the detection alone. The depth of the pothole was used as a profile metric, and the accelerometer data collected by a smartphone were used to conduct the analysis together with the GPS data. The authors of [8] used four different vehicles to collect the data with two samplings rate of 100 and 200 Hz. Twenty-three different types of potholes were considered on an overall set of 2760 segments. In comparison to [8], this paper uses a larger set of vehicles (12), a similar set of road anomalies/obstacles (19), and different sampling rates (50, 100, 150, 200 and 250 Hz). In addition, this paper proposes a deep learning approach, while the analysis in [8] was done with Cumulative Distribution Functions (CDFs). On the other hand, the authors of [8] also investigated different placements of the smartphone in the vehicle with the result that the placement on the dashboard provided the best identification accuracy. Based on the results of [8], this paper adopts the position of the smartphone on the dashboard as well.

The authors of [15] used a DL approach based on CNN (as in this paper) to perform the detection of road anomalies. The results provided in [15] confirmed the superior performance of the adoption of CNN in comparison to shallow machine learning algorithms, which prompted the authors of this paper to apply a CNN based approach as well. In comparison to the CNN approach used by the authors of [15], where the CNN was applied directly to the data collected from the accelerometers, this paper first transforms the accelerometer data in the spectral domain using the spectrogram defined in Section 3.4, then the spectral representation is fed to a CNN (called CNN-SP in the rest of this paper). The results presented in this paper show that CNN-SP outperforms the application of CNN directly on the source data (called CNN-1D in the rest of this paper) both for accelerometer and gyroscope data and across different sampling rates. The application of CNN-SP is evaluated using an experimental dataset collected by the authors through many hours of driving on a realistic road path with various types of road anomalies. The study results presented in this paper use the dataset from [16], where it was used for the different goal of automotive vehicle authentication. Additional details on the dataset are presented in Section 3.

In [17], the authors used both accelerometers and gyroscopes like the study done in this paper to detect speed bumps. The authors applied a number of features to the data recorded by the accelerometer and gyroscope mounted on the vehicle. Then, a genetic algorithm was used to find a logistic model that accurately detects road abnormalities. The results were quite encouraging as the approach was able to achieve an accuracy of 0.9714 in a blind evaluation.

In [9], the authors proposed a road anomaly detection approach based on the application of three different DL algorithms: Deep Feedforward Network (DFN), Convolutional Neural Network (CNN), and Recurrent Neural Network (RNN), and the results were compared. The results showed that the application of DL algorithms was very effective in detecting road anomalies, thus proving the approach also used in this paper. In comparison to this paper, the authors in [9] used a larger set of signals beyond the accelerometers and gyroscopes used in this paper since they also included the shock responses of the four absorbers and the rotation speed. From this point of view, reference [9] represents a significant progress in comparison to the literature. The authors of [9] also compared three different DL algorithms. On the other hand, this paper uses a set of 12 vehicles in comparison to the single vehicle used in [9]. This paper also evaluates the impact of different samples rates on the identification accuracy, while [9] used only the sampling rate of 100 Hz. Finally, this paper uses a time-frequency CNN approach rather than the direct application of DL to the signals in the time domain.

Next, we summarize the progress introduced by this paper in comparison to the references identified in the previous paragraphs. As shown in the results provided in this paper, the time-frequency CNNs provide a superior classification performance to the direct application of CNN to the original time domain signal, as proposed in the literature [9,15]. In comparison to the literature [8,9,13,14,15,17], which used only one or two vehicles (with the exception of [8], where four vehicles were used), this paper uses a relatively large set of vehicles (12) to improve the generalization of the results. Then, the focus of the paper is to investigate how accelerometer and gyroscope readings collected from different vehicles are used to detect road anomalies and obstacles. The impact of various speeds by different vehicles is also mitigated to support the application of time-frequency CNN. In comparison to the literature, this study uses the data both from the accelerometers and the gyroscopes, which was adopted only by a few studies [9,17], as the accelerometer data were generally used [15]. Finally, this study provides a more extensive, to the knowledge of the authors, evaluation of the different sampling rates on the identification accuracy.

## 3. Materials and Methods

### 3.1. Materials

As mentioned before, the dataset used for this experiment consisted of 12 different cars. The specifications of the vehicles are listed in Table 1. The same driver and co-driver were present in every car during the experimental data collection to minimize the potential bias of the driving behavior. Even if it is acknowledged that in practical operations, the co-driver may be different or may be absent, we wanted to limit the number of variables in the study. Future developments will investigate the presence of different passengers and their number (see Section 5). For the IMU, we used the microelectromechanical system based motion tracker supplied by Xsens (Enschede, The Netherlands) with Model Number MTi−100−2A8G4. The technical specification of the Xsens sensor are reported in Table 2.

**Figure 1:**
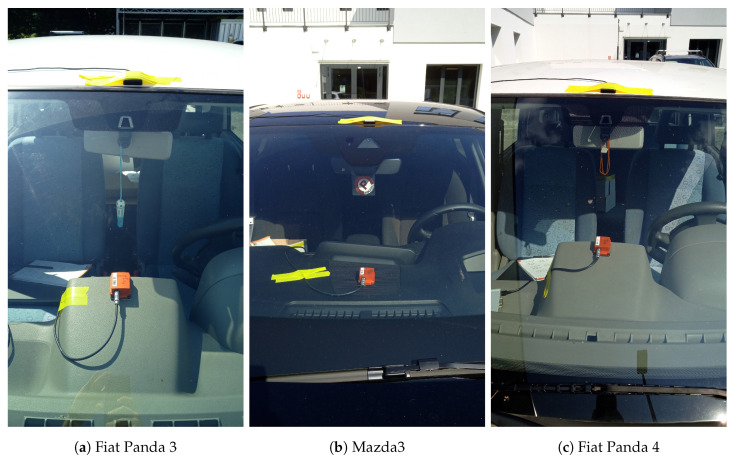
Placement of the sensor used for the data collection in the vehicles for three vehicle models.

The Xsens MTi−100 sensor used for data collection is designed to measure the three axis acceleration and rate of turn at a 2000 Hz sampling rate and the three axis magnetic field at a 100 Hz sampling rate. The actual sampling rate used in the analysis was actually smaller to emulate the sampling rate from a smartphone. In particular, increasing sampling rates of 50, 100, 150, 200 and 250 Hz were used, as this is the range from low cost phones to more sophisticated phones. The IMU was mounted using a strong double sided foam tape at the same spot and orientation for every car. We decided to place the sensor on the top of car’s dashboard in the middle of the car, because it is a common placement position for the IMU in the literature [12]. In the recent review by Menegazzo et al. [4], the placement of the IMU on the top of car’s dashboard was the one mostly adopted in the literature. The results from [8] also showed that the placement on the dashboard provided the smallest classification error in comparison to the placement of the IMU in other locations on the vehicles (e.g., left or right side of the car). The image of the placement of the IMU in three vehicles is shown in Figure 1.

**Figure 2:**
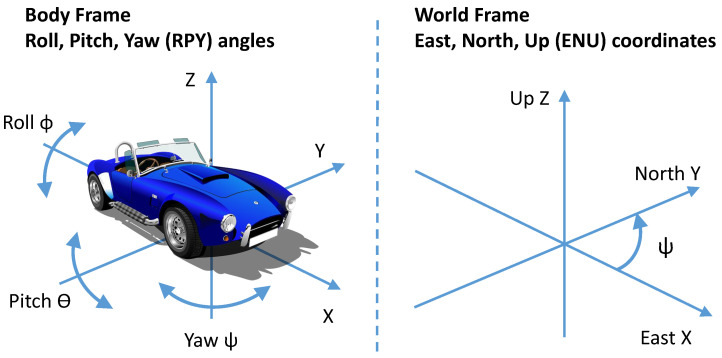
Reference frames.

The data collection was performed using the controlled positioning strategy. As described in [4], this strategy consists of a simple technique where the sensor is placed on the vehicle so that the axes in both reference frames coincide, i.e., the sensor axes are aligned with the vehicle axes, and it is not necessary to apply preprocessing for reorientation. This technique was used for both accelerometer and gyroscope data. Then, the *x*-axis of the IMU was always pointing towards the driving direction and the *z*-axis in the vertical direction. A description of the reference frames used in this paper is shown in Figure 2 with a depiction of the body frame and the world frame. The data collection and analysis were performed on all three axes’ data both for the accelerometers and gyroscopes, but the accelerometer *Z* and the gyroscope *Y* provided the optimal classification accuracy.

The description of the brand and model of each of the 12 cars is shown in Table 1. The models were chosen to have a significant number of cars of the same model (the Fiat Panda), but to include as well car models (Mazda3, Octavia) that are different from the Fiat Panda regarding the weight and engine power. The use of 12 different vehicles mitigated the problem of defining a model for pothole detection based on a single vehicle because the data collected by each vehicle were shuffled in the classification process.

**Figure 3:**
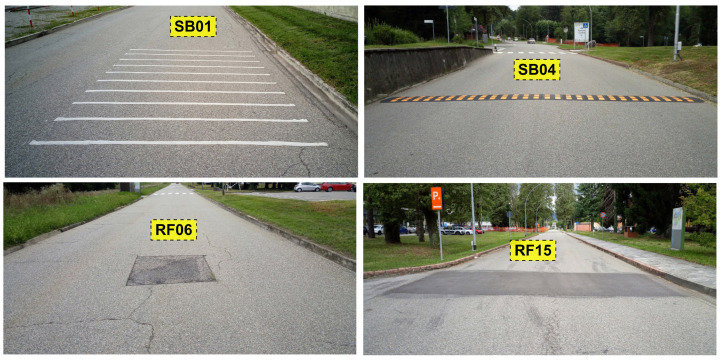
Examples of obstacles and road anomalies (picture).

**Figure 4:**
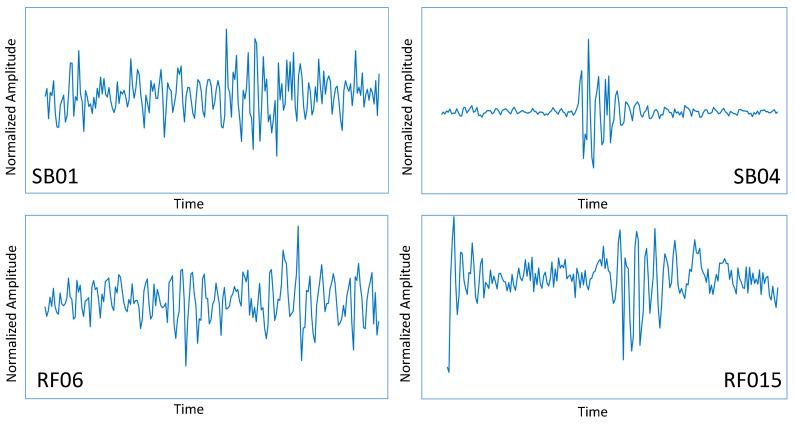
Examples of obstacles and road anomalies (recordings with the accelerometer in the Z vertical direction).

The path where the vehicles were driving was a loop in the European Commission Joint Research Centre (JRC) premises. The path is slightly longer than 2 km. Since the data collection was performed for each of the 12 vehicles driving 20 times on the loop, the entire driving data collection was performed on more than 480 km of road. The JRC campus was built in 1960–1965, and the creation of the road infrastructure was more than 50 years old at the time of writing this paper. The road infrastructure is well maintained, and it is all asphalted, even if the asphalt is relatively old and presents various road anomalies like potholes, cracks, transverse cracks, and patches. Different parts of the loop also have different maintenance records because the loop crosses different sectors of the JRC campus. Then, the road surface condition is uneven across the different parts of the loop. The loop includes 15 main road anomalies and 4 obstacles of two different types, which were selected for the data analysis. With reference to the classification of exteroceptions identified in [4], the road anomalies included potholes, cracks, transverse cracks, and patches, while the obstacles were of types rumble strips and speed humps/speed bumps. A picture of some examples of the speed bumps and the road anomalies (potholes in the road) are shown in Figure 3 with the related recordings with the accelerometer in the *Z* direction in Figure 4.

**Figure 5:**
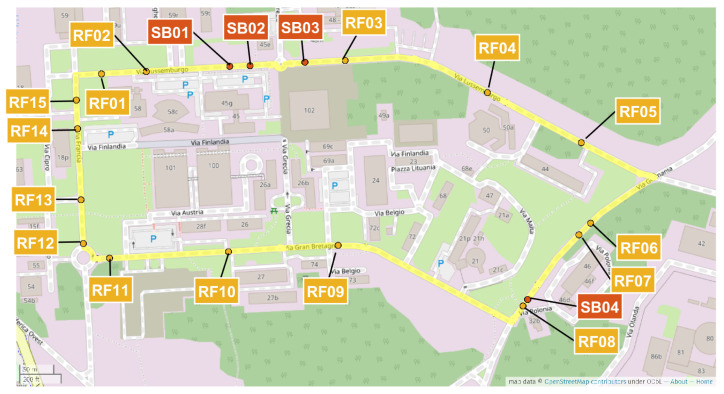
Map of the driving path/loop with the position of the road anomalies/obstacles.

The road anomalies are identified in the rest of the paper with the identifier RFX with X = 01, …, 15 while the obstacles are identified with SBY with Y = 01, …, 04. The segments of the loop, which are outside RFX and SBY, are identified as NORMZ with Z = 01, …, 22. See Section 3.2 for further details. Note that even the NORM road segments are not exempt from the presence of small road anomalies or uneven surfaces. The map with the pictorial description of the driving path and the position of the road anomalies/obstacles is shown in Figure 5.

### 3.2. Methodology

**Figure 6:**
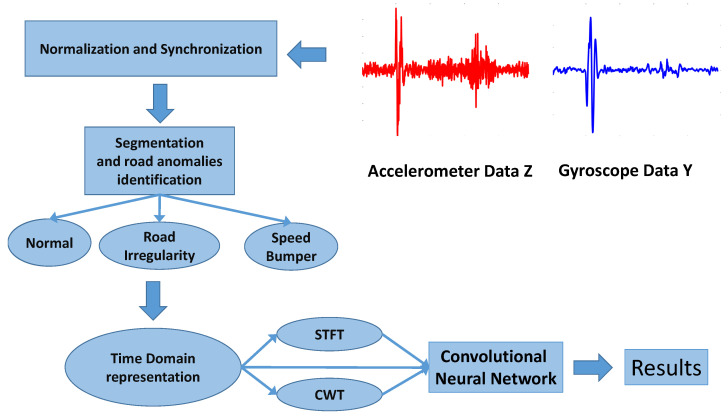
Overall methodology.

The overall methodology is described in Figure 6, and each step is described in the following paragraphs:

- Normalization and synchronization: As a first step, the data collected from the IMU in each of the 12 vehicles were synchronized among the 20 laps and normalized. A GNSS receiver was also installed in the vehicle and synchronized with the IMU. This process was repeated for different sampling rates: 50, 100, 150, 200 and 250 Hz. All these sampling rates were obtained by downsampling by the related factor (e.g., a factor of 10 for 200 Hz) the initial data collected at 2000 Hz from the IMU. In this paper, we consider only the analysis of the accelerometer data in the *Z* direction (the vertical direction) and the gyroscope in the *Y* direction (in the direction of the vehicle). The reason for this choice was to minimize the degrees of freedoms in the analysis and because a heuristic analysis of the data related to the other axes of the accelerometers and gyroscopes showed that the obtained accuracy was inferior to the one obtained using the accelerometer in the *Z* direction and the gyroscope in the *Y* direction. This is to expected because the IMUs were mostly stimulated for those axes by the roughness of the road surface, and this is also consistent with literature [11]. In the rest of this paper, the Accelerometer data in the Z direction is called AccZ, and the Gyroscope data in the *Y* direction is called GyroY. The relationship between the RPY angles and the ENU coordinates was the same as described in [16]. The synchronization was performed by applying the moving variance to the data from the accelerometer and by correlating the results across the laps and the vehicle. As is well known in the literature, full synchronization is not always possible because the vehicles move at different speeds over the road anomalies, and each vehicle has its response to the stimulus created by the road anomaly. On the other side, these are problems derived from data collection in a realistic environment, and such processing issues are likely to be present in any real scenario.
- The entire loop was divided into three different types of segments, and each segment was labeled with type normal, road anomaly identifier (e.g., an asphalt break is RF15), and obstacle identifier (e.g., SB01). There were 4 obstacles (identified with SB01, SB02, SB03 and SB04), 15 road anomalies or road features (identified with RFX and X = 01, …, 15), and 22 normal segments (identified with NORM), which were obviously the majority in the entire loop. Each segment had a time duration of 4 s. This time duration was chosen because it was long enough to include the driving time of a vehicle over each of the road anomalies/obstacles considered in the study. The approach was tested on different driving speeds since each vehicle (of the dataset of 12 vehicles) was driving at a different speed and the speed was different in each loop (20 loops), even for the same vehicle. Then, the data collection was representative of the real-world conditions when vehicle speeds may be different. Indeed, this was the focus of the study to evaluate if the proposed approach was able to compensate the data taken with different speeds. As the segment duration was fixed, the sample length of each segment was obviously longer for higher sampling rates (e.g., a segment was 200 samples long for a sampling rate of 50 Hz). The labels were generated by using the GNSS position and by manually checking for each segment that the road anomaly and the obstacle were correctly assigned to each segment. This manual step was needed because the GNSS accuracy may not be precise enough to identify the precise location of the road anomaly/feature.
  As we had 12 vehicles for 20 loops, the analysis took into consideration a total of 12 × 20 × 41 segments = 9840 segments based on (4 + 15 + 1) = 20 different classes.
- The spectrogram was applied to the time domain digital output from the IMU (AccZ and GyroY) for the considered sampling rates and by using different values of the hyperparameter. The definition of the spectrogram and its hyperparameters is provided in Section 3.4.
- The segments of the different representations were used as an input to a CNN, which is described in Section 3.3. The initial time segment was also used as the input to three machine learning algorithms, also described in Section 3.3. We note that all the data measurements from the 12 vehicles were used for classification. Then, the classification using CNN was performed on a model based on the data from all 12 vehicles, which enhanced the generalization of the proposed approach in comparison to models based on the data collected by a single vehicle.

### 3.3. Machine Learning

As mentioned before, three different machine learning algorithms were used, and the results were compared: a CNN using different representations, Support Vector Machine (SVM), and K Nearest Neighbor (KNN). Each machine learning algorithm was based on a set of hyperparameters and their optimal values, which are summarized in Table 3. The following paragraphs describe each specific algorithm and how the hyperparameter values were identified. All the machine learning algorithms were implemented in MATLAB. The three machine learning algorithms were chosen on the basis of the following considerations.

**Figure 7:**
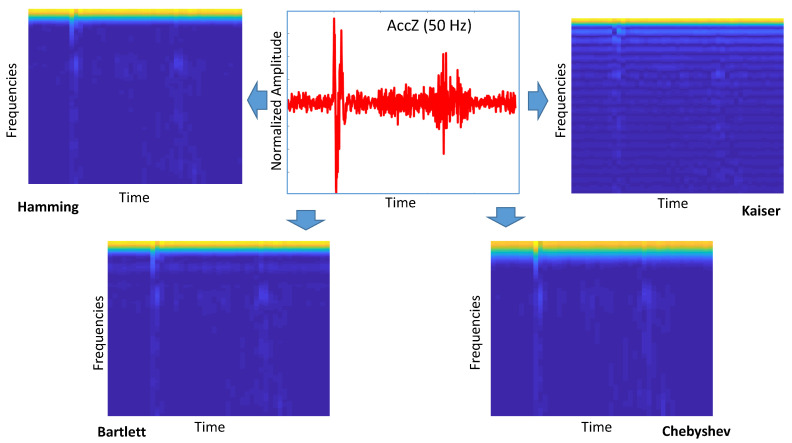
Application of the spectrogram to the Accelerometer Z (AccZ) output from the IMU.

**Figure 8:**
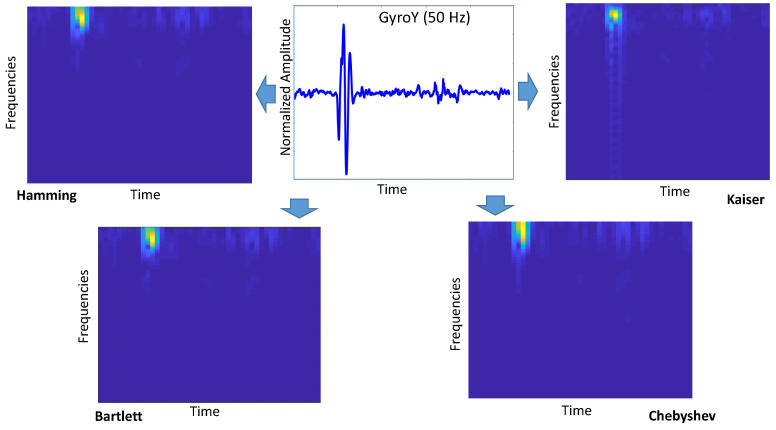
Application of the spectrogram to the Gyroscope Y (GyroY) output from the IMU.

The main objective of this study was to investigate the performance of Deep Learning (DL) for this particular problem of the identification of road anomalies and obstacles. Among other DL algorithms, CNN has been widely applied to image processing since the excellent results from the ImageNet Large Scale Visual Recognition Competition (ILSVRC) in 2012 [18]. The approach proposed in this paper is based on the transformation to images of the accelerometer/gyroscope readings in the time domain collected on the vehicles while driving (see Figure 7 and Figure 8). The intimate relationship between the layers and spatial information in CNNs renders them well suited for image processing and for extracting the discriminating characteristics of the road anomalies and obstacles [19].

**Figure 9:**
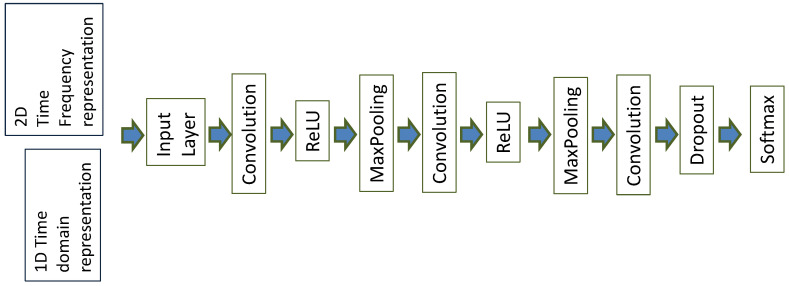
CNN architectureused for the classification.

The relatively small size of the dataset (9600 segments of road) for deep learning may generate the risk of overfitting. This risk was mitigated by tuning the hyperparameters (e.g., *L2*) to improve generalization, by using a dropout layer (see Figure 9) and by using cross-validation using 12 folds, as described in the subsequent paragraphs. We also highlight that the number of segments was comparable to the ones used in the literature where deep learning was also applied [9,15].

Then, two shallow machine learning algorithms were used to compare their classification performance to the deep learning CNN algorithm described above. In particular, SVM and KNN were used. The SVM is a computational learning method based on statistical learning theory, whose algorithm constructs and then searches the separating hyperplanes with the maximum margin by transforming the problem description into the dual space by means of the Lagrangian. SVM is reported to be successfully applied in many classification problems (object detection and recognition, information and image retrieval) with a good generalization ability [20]. SVM can deliver a unique solution, since the optimality problem is convex, while other algorithms (like neural networks) may have multiple solutions associated with local minima. In addition, SVM is well known for its effectiveness in high-dimensional spaces, as in the dataset used in this study.

The SVM was also used since it is widely adopted in the literature for road anomaly detection when a machine learning approach is used. The survey presented in [4] for the machine learning algorithms showed that SVM is the most used machine learning algorithm and the second in the overall ranking (the first approach in the ranking is the threshold based approach, which is not a machine learning algorithm and, therefore, is not adopted in this paper).

The KNN was chosen as a baseline for comparison with the other two algorithms as it is relatively simple and naturally lends itself to multi-class problems, as in this case.

The architecture of the CNN is shown in Figure 9, and a brief description is provided here. The input layer of the CNN depended on the type of adopted transformation (e.g., in the 1D time domain, the representation is 1 × 200). The convolutional layers’ parameters (e.g., stride) were optimized according to the specific input, as this input changes for the different representations (e.g., time or spectrogram). Padding was used. The number of filters was set to 30 (an analysis range between 20 and 40 was considered), and the max pooling was set to 4. The solver RMSProp was used as it provided a superior performance to other solvers (stochastic gradient descent and stochastic gradient descent with momentum) for this specific dataset with a learning rate of 0.001 (optimization range between 0.0001 and 0.01). The batch size was set to 128. The number of epochs was set to 160. To mitigate overfitting, the *L2* parameter was set to 0.0005 (optimization range between 0.0001 and 0.01), and a dropout layer was used. Cross-entropy was used as the loss function. The Number of Convolutional layers (*NC*) was also optimized in the range *NC* = 2 to *NC* = 5. The optimal value for the identification accuracy of *NC* was determined to be *NC* = 3, which defines the architecture described in Figure 9. Because the number of hyperparameters to optimize was significant (number of filters, solver, *L2*, batch size, and number of convolutional layers), each parameter was optimized in a sequential way by keeping fixed the value of some parameters and performing the optimization on other parameters. The first parameter, to be optimized, was the number of convolutional layers *NC* (i.e., the CNN architecture) with *Nf* = 20, solver = SGD, *L2* = 0.001, and batch size = 64. Then, the solver parameter was optimized with the identified number of convolutional layers (i.e., *NC* = 3). Finally, a three-dimensional grid approach was used to select the optimal values of the parameters *Nf*, *L2*, and batch size. The resulting values from the optimization process are presented in Table 3.

SVM is a supervised learning model that classifies data by creating a hyperplane or set of hyperplanes in a high- or infinite-dimensional space, to distinguish the samples belonging to different classes. As this paper addresses a multi-class machine learning problem, a multi-class SVM was used, which was based on an Error-Correcting Output Coding (ECOC) classifier for multiclass learning, where the classifier consists of multiple binary learners. In particular, we used the OneVsOne approach where for each binary learner, one class is positive, another is negative, and the algorithm ignores the rest. This design exhausts all combinations of class pair assignments. Various kernels (e.g., linear, polynomial) were tried, and the one providing the best performance was the Radial Basis Function (RBF) kernel, where the values of the scaling factor γ must be optimized together with the parameter *C* [21]. In this paper, the SVM was applied with the RBF with a scaling factor $\gamma =2^{5}$ and a $C_{factor}=2^{7}$. The optimization was performed on a range of values for γ and *C* between $2^{2}$ and $2^{10}$ using a grid search approach. The optimal values of the parameters are presented in Table 3.

K Nearest Neighbor (KNN) is an approach to data classification that estimates how likely a data point is to be a member of one class or another depending in which group the data points nearest to it are. KNN is an example of a lazy learner algorithm, meaning that it does not build a model using the training set until a query of the dataset is performed. The main hyperparameter in KNN is the *K* factor, which must be optimized for the specific classification problem. The type of distance metric used to calculate the “nearest” must also be chosen carefully [22]. The optimization was performed on a range of values of *K* from 1 and 10 for each of the different distance: Euclidean, Manhattan, Chebyschev, and Mahalanobis. The optimal values of the hyperparameters, which provided the highest accuracy, are listed in Table 3.

All the optimized values presented in this section were calculated using a linear or grid approach for each sampling rate and then averaged for the analysis of the time domain data.

For all three machine learning algorithms, a 12-fold cross-validationwas used with the partition of the entire set into 12 exclusive folds. For each fold, eleven of twelve parts of the initial dataset were used for the training plus validation, while the remaining 1/12 part was used for testing. The validation part was 1/4 of the training plus validation portion (thus, the validation part was 11/48 of the entire dataset in each fold). Then, the results were averaged.

The evaluation metrics were the accuracy, precision, and recall, and they are described in the following equations:

(1) $$Accuracy=\frac{\left(TP+TN\right)}{\left(TP+TN+FP+FN\right)}$$

(2) $$Precision=\frac{\left(TP\right)}{\left(TP+FP\right)}$$

(3) $$Recall=\frac{\left(TP\right)}{\left(TP+FN\right)}$$

where *TP* is the number of True Positives, *FP* is the number of False Positives, *FN* is the number of False Negatives, and *TN* is the number of True Negatives.

### 3.4. Time Frequency Transforms

This section is focused on the description of the spectrogram and the related hyperparameters. In addition, because Section 4 provides a comparison with another time-frequency representation based on ContinuousWavelet Transform (CWT), the CWT definition and hyperparameters are also described here.

Consider a signal s(τ) of length *N* (the original signal in the time domain, called 1D-T in the rest of this paper) and a window w(τ) of length $W_{size}$ (where $N>>W_{size}$), whose Fourier transforms are respectively *S(f)* and *W(f)*, as shown in the following equations:

(4) $$S\left(f\right)=\int_{-\infty }^{\infty }s\left(\tau \right)e^{-j2\pi f\tau }d\tau$$

(5) $$W\left(f\right)=\int_{-\infty }^{\infty }w\left(\tau \right)e^{-j2\pi f\tau }d\tau$$

The localized spectrum representation of s(τ) at time τ=t is obtained by multiplying the signal by the window w(τ) centered in time τ=t, as shown in the following equation:

(6) $$s_{w}\left(t,\tau \right)=s\left(\tau \right)w\left(\tau -t\right),$$

through the application of the FT, the Short Time Fourier Transform (STFT) $F_{s}^{w}\left(t,f\right)$ is obtained:

(7) $$F_{s}^{w}\left(t,f\right)=F_{\tau \to f}\left\{s(\tau )w(\tau -t)\right\}$$

The squared magnitude of the STFT, denoted by $S_{s}^{w}\left(t,f\right)$, is called the spectrogram and is expressed by the following equation:

(8) $$S_{s}^{w}\left(t,f\right)={\left|F_{s}^{w}\left(t,f\right)\right|}^{2}$$

As described in Section 4, the spectrogram was adopted for the analysis presented in this paper because it provided a superior identification performance in comparison to the phase component of the STFT, as shown in Table 4.

In the spectrogram definition, we evaluated the impact of three main hyperparameters: (1) the type of window w(τ), (2) the length of the window or window size $W_{size}$, and (3) how much overlapping was between different windows on the overall length *N* of the signal x(τ). The overlapping is defined as $O_{lap}$, and it is defined as a percentage of the window size $W_{size}$ while the window is sliding across x(τ). Because the parameter $W_{size}$ is dependent on the sample ratio at which the data are collected by the IMU and it would not be the same across different sampling rates, the parameter $W_{R}$ is instead used in the rest of this paper. $W_{R}$ is defined as the ratio of *N*/$W_{size}$. For example, a value of $W_{R}$ = 10 at a sampling rate of 50 Hz results in $W_{size}=20$.

Four different window types were used: Hamming, Bartlett, Chebyschev, and Kaiser [23], and they were chosen because of their different filtering behaviors. Three different window sizes with different values of $W_{R}$ were identified: $W_{R}$ = 4, 5, and 10. Finally, three different overlapping factors $O_{lap}$ were evaluated: 33%, 50%, and 66%. The results of the evaluation are presented in the next Section 4.

The other time-frequency representation was based on the wavelets. The CWT provides an overcomplete representation of a signal by letting the translation and scale parameter of the wavelets vary continuously [24].

The CWT is expressed by:

(9) $$C\left(a,b\right)=\frac{1}{{\left|a\right|}^{1/2}}\int_{-\infty }^{\infty }s\left(\tau \right)\psi \left(\frac{\left(\tau -b\right)}{a}\right)d\tau$$

where ψ is the mother wavelet, a is the scale, and b is the translational value. In this analysis, we chose the Morse mother wavelet [25] and the CWT implementation in MATLAB from MathWorks (i.e., the cwt function).

## 4. Results

In an initial step, the impact of the hyperparameters of the spectrogram definitions on the identification accuracy was evaluated.

### 4.1. Optimization of the Spectrogram Hyperparameters

This section is focused on the optimization of the hyperparameters in the spectrogram.

**Figure 10:**
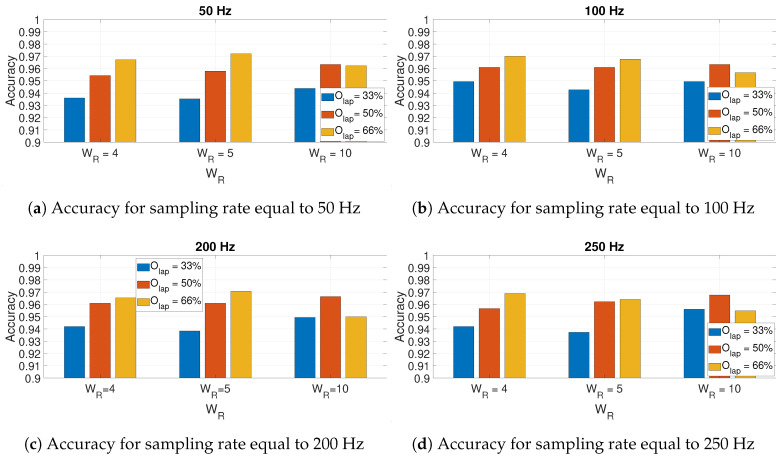
Accuracy for AccZ in relation to the size of window (based on the *W_R_* factor) used in the spectrogram for different sampling rates and different values of the overlapping factor *O_lap_*. The Hamming window is used to obtain these results.

**Figure 11:**
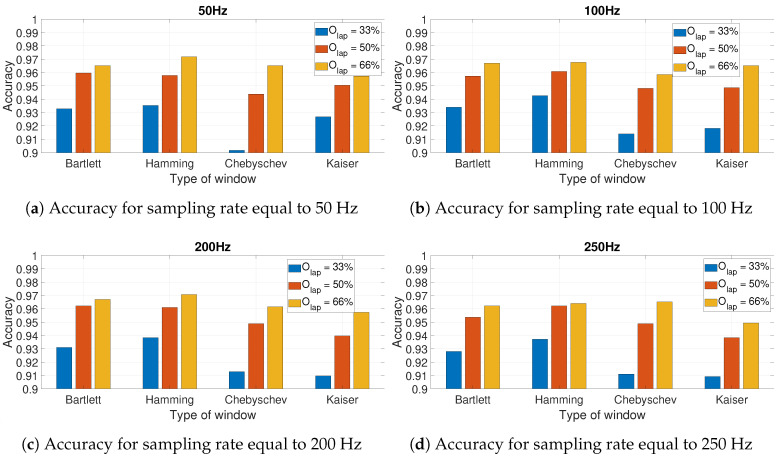
Accuracy for AccZ in relation to the type of window used in the spectrogram for different sampling rates and different values of the overlapping factor *O_lap_* and *W_R_* = 5.

Figure 10 presents the results for the calculated accuracy using the CNN-SP approach using AccZ. The results are presented as a bar graph with different bars related to the hyperparameters of the spectrogram definition including the size of the window (based on the $W_{R}$ factor) and the different values of the overlapping factor $O_{lap}$. Four different graphs are presented for the different sampling rates: 50 Hz (Figure 10a), 100 Hz (Figure 10b), 200 Hz (Figure 10c), and 250 Hz (Figure 10d). The sampling rate of 150 Hz is not presented because of the lack of space and because it is quite similar to other graphs. The graphs in Figure 10 shows that for $W_{R}=4$ and $W_{R}=5$, the highest accuracy was obtained with $O_{lap}=66\%$, while for $W_{R}=10$, the highest accuracy was obtained with $W_{R}=10$. On the other side, it is noted that for most of the shown sampling rates (e.g., 50, 100 and 200 Hz), the accuracy obtained with $W_{R}=10$ was the lowest of all the values of $O_{lap}$, while this was different for the sampling rate of 250 Hz. The use of higher sampling rates also increased the classification time; then, if there is no loss of classification accuracy (as shown in Figure 10), it is preferable to use the optimal parameters for the lower sampling rate (i.e., 50 Hz). In this case, such an optimal value was obtained with $W_{R}=5$ and $O_{lap}=66\%$. These results were obtained with the Hamming window. The choice of this window is supported by the results presented in Figure 11 for different windows and overlap values $O_{lap}$ with $W_{R}=5$. In fact, the results depicted in the figures below show that the optimal identification accuracy is obtained with the Hamming window across all the sampling rates and the $O_{lap}$ values, and this is the type of window chosen in the subsequent results.

**Figure 12:**
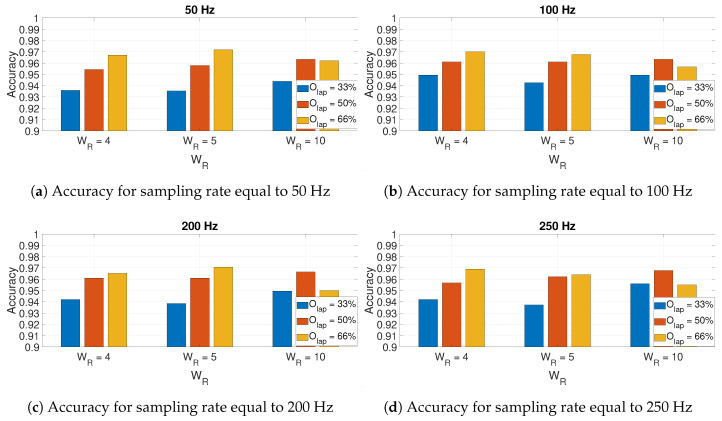
Accuracy for GyroY in relation to the size of window (based on the *W_R_* factor) used in the spectrogram for different sampling rates and different values of the overlapping factor *O_lap_*. The Hamming window is used to obtain these results.

**Figure 13:**
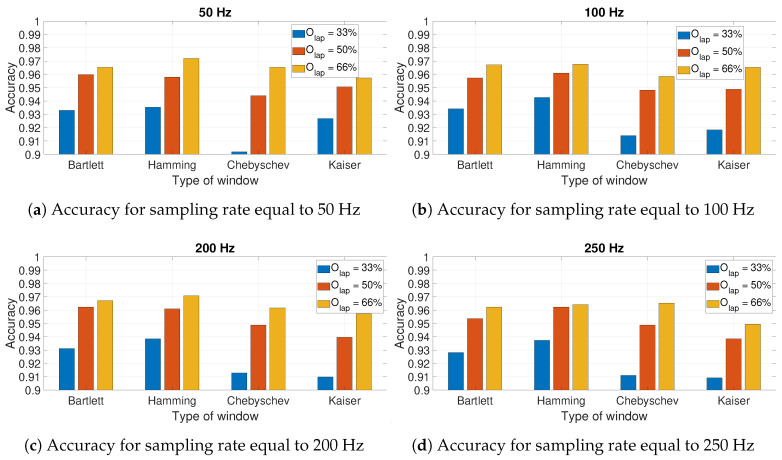
Accuracy for GyroY in relation to the type of window used in the spectrogram for different sample rates and different values of the overlapping factor *O_lap_* and *W_R_* = 5.

The results obtained for GyroY are shown in Figure 12. In general, the results for GyroY confirm the previous results obtained with AccZ, but with greater variability. In fact, $O_{lap}=66\%$ provides better results for the sampling rate of 100 Hz (Figure 12b) and the sampling rate of 200 Hz (Figure 12c) for $W_{R}=4$ and $W_{R}=5$, but in other cases, $O_{lap}=50\%$ provides better results. We highlight that the highest classification accuracy (i.e., reaching the threshold of 0.97) is quite similar to the one obtained with AccZ. These results indicate that the use of AccZ or GyroY provides an equivalent classification accuracy. All these results are obtained with the Hamming window. As shown in Figure 13, the Hamming window provides better results than the other windows across all the different sampling rates. From this point of view, these results obtained for the gyroscope are consistent with the results obtained with the accelerometer.

### 4.2. Analysis and Comparison of the Approaches

**Figure 14:**
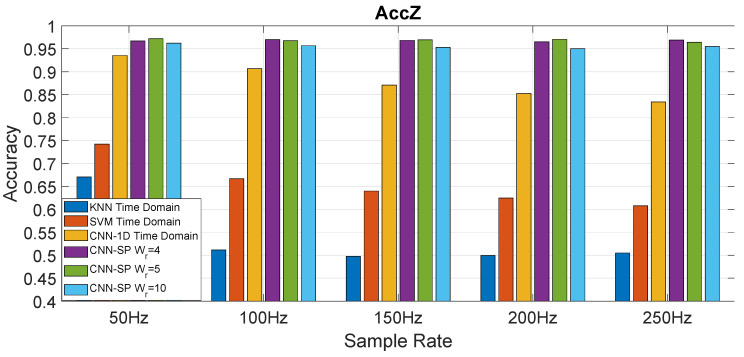
Comparison of the accuracy among the spectrogram CNN approach (CNN-SP) proposed in this paper (for different window sizes), the CNN directly applied to the time domain representation (CNN-1D), and the shallow machine learning techniques (SVM, KNN) applied to the data of the Accelerometer in the *Z* direction (AccZ).

**Figure 15:**
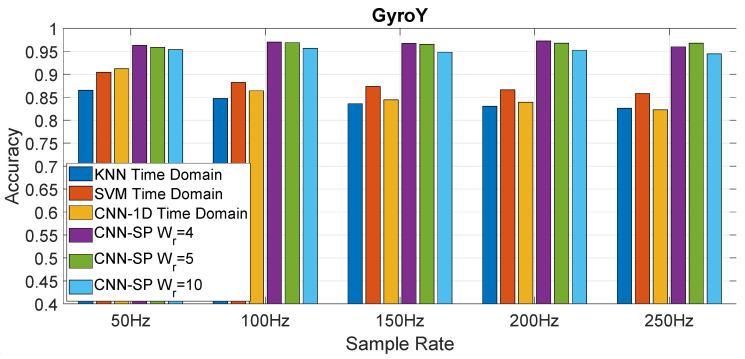
Comparison of the accuracy among the spectrogram CNN approach (CNN-SP) proposed in this paper (for different window sizes), the CNN directly applied to the time domain representation (CNN-1D), and the shallow machine learning techniques (SVM, KNN) applied to the data of the Gyroscope in the *Y* direction (GyroY).

This section presents the results of the comparison of the proposed approach with the shallow machine learning algorithms and CNN-1D. Figure 14 provides the comparison of all the different approaches evaluated in the analysis for the accelerometer in the *Z* direction (AccZ), while Figure 14 provides the comparison for the gyroscope in the *Y* direction (GyroY). As mentioned before, CNN-SP is the application of CNN to the spectrogram of the original signal in the time domain; CNN-1D is the application of the CNN to 1D-T (the original signal in the time domain). SVM and KNN represent the direct application of the respective shallow machine learning algorithm to 1D-T. The presented results are based on the mean (average) of the results calculated for each fold across the 12 folds. The results clearly show that all the CNN based approaches significantly outperform the shallow machine learning approach in a consistent way across all the different sampling rates, which confirms the results obtained by the authors in [15] for a different dataset. In addition, the approach proposed in this paper based on the combination of the CNN with the spectrogram (CNN-SP) outperforms in a consistent way the direct application of CNN on the time domain (CNN-1D), which is the novel finding of this paper. The other interesting results provided by Figure 14 and Figure 15 are that the CNN-SP approach provides a relatively uniform value of the accuracy across the different sampling rates, while CNN-1D drops significantly with higher sampling rates. A potential reason for this behavior is that the CNN algorithm is able to extract similar discriminating features across the different sampling rates when applied in combination with the spectrogram, while lower sampling rates in 1D-T introduce a smoothing effect in CNN-1D, which benefits the classification accuracy. A similar consideration can be applied to the application of the SVM and KNN algorithms, whose classification accuracy also degrades with increasing sampling rates.

For completeness, we also report the accuracy obtained for another time-frequency representation as mentioned previously (i.e., the CWT) and for the application of the STFT in phase (the complex amplitude of the STFT is the spectrogram already considered before). The results are shown in Table 4. Note that the results presented for the STFT phase and the CWT are the result of an optimization process similar to what was done for the spectrogram in Section 4.1. For the CWT, this was done on the type of the wavelet and the number of octaves for frequency. For the STFT (phase), this was based on the same parameters identified in Section 4.1. The results clearly confirm that CNN-SP provides a superior classification performance in comparison to the other approaches. In particular, the use of the magnitude-only components of CNN-SP provides a better identification accuracy than the phase-only component (and the combination of amplitude plus phase, which is provided here). The other spectral approach based on the use of CWT also provides an inferior performance, which indicates that the wavelets may not be appropriate for this context (note that a similar result for vehicle authentication was also obtained in [16]).

The accuracy metric only provides a limited view of the classification performance. For this reason, we also provide in the following paragraphs and figures the estimate of the recall and precision obtained for the specific set of parameters and for different machine learning algorithms. The following figures show the recall and precision for each specific road anomaly when compared to the other anomalies, and they give a better understanding of the areas where the machine learning failed to identify the correct samples (false positives and false negatives).

**Figure 16:**
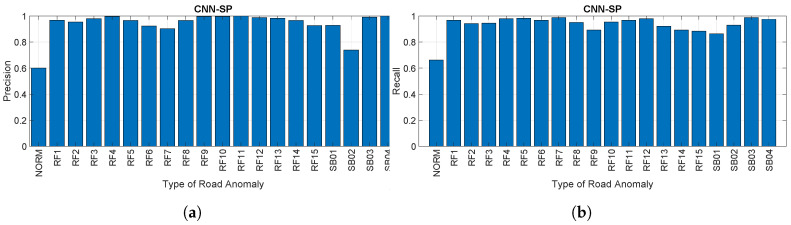
Precision and Recall using the *CNN* − *SP* approach. (**a**) Precision for each road anomaly for CNN-SP (sampling rate of 50 Hz, *O_lap_* = 66%, *W_R_* = 5) and (**b**) recall for each road anomaly for CNN-SP (sampling rate of 50 Hz, *O_lap_* = 66%, *W_R_* = 5).

Figure 16a,b provides respectively the precision and recall for each road anomaly as a percentage using the CNN-SP approach with a sampling rate of 50 Hz, $O_{lap}=66\%$, and $W_{R}=5$. Similar results were obtained for the other values of the CNN-SP hyperparameters, but they are provided here for space reasons. These figures confirm the previous findings on the accuracy, which shows that it is possible to obtain a great identification accuracy using the CNN-SP approach. On the other hand, the figures show that there can be great variability for the identification of each road anomaly/obstacle. Of course, this depends on the type of road anomaly/obstacle and how much different from the other road anomalies/obstacles it is. One relevant aspect is the relatively low value of recall and precision for the class NORM. The reason is that the machine learning algorithm confuses in some cases the road anomaly/obstacle with the normal segment (identified as NORM in the figures), because the digital output from the accelerometers/gyroscopes is similar. In fact, even a NORM segment includes some small irregularities (e.g., cracks, uneven condition of the road), which may be difficult to distinguish. This result is to be expected, and it is consistent with the findings in the literature [15].

On the other side, such low values of precision and recall may be a significant problem when used in real-world applications since most real-world road segments are normal. A potential reason for these results is that no thresholds were applied for the detection of road anomalies/obstacles, but the machine learning algorithms were directly applied to the data recordings. Different approaches can be used to mitigate this issue. Some of these approaches are based on a similar analysis from previous studies in the literature, and they can be classified into different phases of the overall methodology: data collection, data pre-processing, or data processing/classification (e.g., based on the ML/DL algorithm). One potential approach in the data collection phase would be to widen the sensor inputs using the shock responses of the four absorbers or the wheel speed from the four wheels, as was done in [9] to enhance the discriminating power of the classification algorithm. Another approach in the data pre-processing is to introduce a smoothing filter in the data pre-processing phase. We note that the evaluation of the identification performance with different sampling rates described in this study is also a crude form of smoothing for the lower sampling rates. A review of the application of filters in the literature for road anomalies’/obstacles’ detection was provided in Section 2.2. of [10]. Another possibility would be to set a threshold to analyze only the most significant road anomalies/obstacles and remove the smaller ones, which are present in the normal segment (see Section 2.3 of [10] for a review on threshold techniques). Note that the thresholds’ definitions do not need to be static, but they can also be dynamic to adapt themselves to the road conditions, as proposed in [26]. The application of both techniques would require significant effort to identify the most effective smoothing filter or the appropriate values of the threshold. For this reason, this analysis is postponed to future developments (see Section 5). In the data processing/classification phase, the existing dataset could be widened using data augmentation techniques (e.g., adding noise) to improve generalization and robustness. Another approach would be to use other time-frequency transforms (e.g., Stockwell transform) in combination with CNN or with other deep learning algorithms.

**Figure 17:**
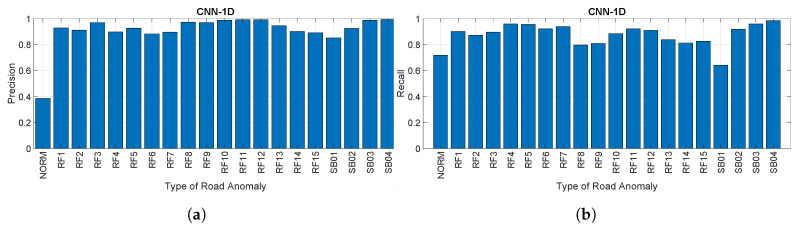
Precision and recall using the CNN-1D approach. (**a**) Precision for each road anomaly for CNN-1D (sampling rate of 50 Hz) and (**b**) recall for each road anomaly for CNN-1D (sampling rate of 50 Hz).

Figure 17a,b provides respectively the precision and recall for each road anomaly as a percentage using the CNN-1D approach at a sampling rate of 50 Hz. The results are similar to what was achieved for the CNN-SP approach, but the recall and precision are slightly lower, as already foreseen from the results presented in Figure 14. In particular, the same phenomenon of a lower value of precision and recall is also noted here.

**Figure 18:**
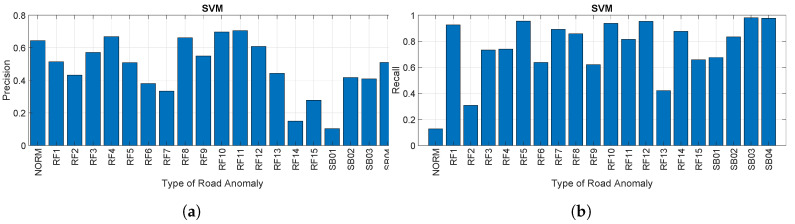
Precision and recall using the SVM algorithm. (**a**) Precision for each road anomaly for SVM (sampling rate of 50 Hz) and (**b**) recall for each road anomaly using SVM (sampling rate of 50 Hz).

Figure 18a,b provides respectively the precision and recall for each road anomaly as a percentage using the SVM algorithm at a sampling rate of 50 Hz. The figures show the drastic drop in the classification performance in comparison to the CNN based approach. In addition, we note a greater variability in the identification of the specific road anomalies among them. In general, the precision is negatively impacted as the number of False Positives (FP) is quite significant. In particular, the algorithm confuses specific road features (e.g., RF14) or obstacles (e.g., SB01 and SB02) as normal road segments (i.e., NORM). In comparison, recall is usually higher for the same road anomalies, which shows that the False Negatives (FN) are more limited in number than the False Positives (FP).

**Figure 19:**
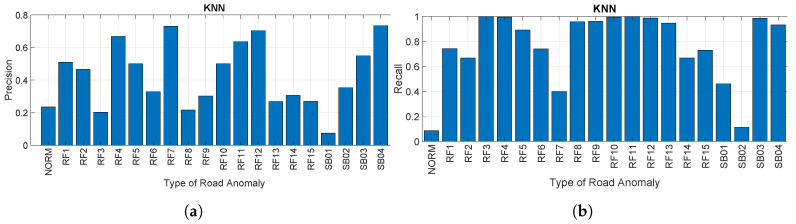
Precision and recall using the KNN algorithm. (**a**) Precision for each road anomaly using KNN (sampling rate of 50 Hz) and (**b**) recall for each road anomaly using KNN (sampling rate of 50 Hz).

Figure 19a,b provides respectively the precision and recall for each road anomaly as a percentage using the KNN algorithm at a sampling rate of 50 Hz. These results confirm what was already obtained for the accuracy. The variability is even increased in comparison to the SVM approach. In addition, the recall for the NORM segment reaches quite low values, which highlights the difficulty of the algorithm to distinguish the NORM segments from the other segments. It is also noted that the lower values of precision and recall are usually for specific road anomalies (e.g., RF14 or SB01), in a similar way to what was obtained for the previous figures and algorithms.

### 4.3. Discussion

The results shown in the previous subsections prove the superiority of the time-frequency CNN (TF-CNN) for road anomaly identification in comparison to shallow machine learning algorithms (SVM, KNN) and to the application of CNN to the initial time domain representation (accelerometer and gyroscope). The difference in classification performance appears for all the considered sampling rates (i.e., 50, 100, 150, 200 and 250 Hz) and both for accelerometer and gyroscope data. With accelerometer data, the improvement in the identification accuracy of TF-CNN is even more visible than with gyroscope data. The final identification accuracy taking into consideration all the road anomalies and the vehicles used in the data collection is slightly more than 97%, which is consistent with the findings in the literature. We note that this value of identification accuracy was obtained by using a set of 12 different vehicles rather than the one or two vehicles commonly used in literature. Then, the high accuracy obtained with TF-CNN is even more remarkable because it manages to mitigate the differences of the mechanical configuration of the vehicles, and it can be used to support crowd-sourcing approaches (with many different vehicles on the road) for the detection and mapping of the road anomalies in the road infrastructure.

Regarding the choice of the time-frequency transform, the results in Table 4 show that the spectrogram in combination with CNN (CNN-SP) provides a slightly better identification accuracy than the application of the complex magnitude of the CWT (CNN-CWT). Additional time-frequency transforms could also be applied, and this will be the scope of future studies (see Section 5).

Another important result from Figure 14 and Figure 15 is that CNN-SP is able to provide a high identification accuracy (higher than 97%) even at lower sampling rates (e.g., 50 or 100 Hz), which supports the deployment of the proposed approach even with cost effective mass-market smartphones, which are now equipped with accelerometers and gyroscopes with a sampling rate of 100 Hz.

We note that the study presented in this paper conducted an extensive analysis of the hyperparameters present in the definition of the time-frequency transforms (e.g., window size, type of window, overlapping ratio) to evaluate their impact on the identification accuracy. The results show that although some hyperparameters do not have a significant impact, other hyperparameters like the overlapping ratio must be carefully tuned in a training phase to achieve the optimal identification accuracy.

One aspect that must still be improved is the relatively low precision and recall of the NORM segments. Potential approaches to mitigate this issue would be to introduce a smoothing filter in the data pre-processing phase, but this would entail an extensive analysis of the choice of the most appropriate filter, so for this reason, this analysis is postponed to future developments (see Section 5).

## 5. Conclusions and Future Developments

This paper proposes a novel approach for the detection and identification of road anomalies using data collected from accelerometers and gyroscopes installed on the vehicles. The approach is based on the transformation of the collected data into the spectral domain (via a spectrogram), which is then given as an input to CNN. This approach is compared against the direct application of CNN on the samples collected in the time domain and on the application of shallow machine learning algorithms like SVM and KNN, as is usually proposed in the existing research literature. This approach is evaluated on a dataset, created by the authors, by collecting IMU (e.g., accelerometers, gyroscopes) recordings on many km of road infrastructure using 12 different automotive vehicles. A comprehensive analysis of the influence of the hyperparameters on the classification performance is presented for the data collected from the accelerometers and gyroscopes. The results show that this approach is able to obtain a very high identification accuracy for each road anomaly, and it is able to distinguish accurately between obstacles intentionally created by road traffic authorities against road anomalies (potholes), which are the consequences of road degradation and roadworks. Beyond the analysis of the road roughness, such high accuracy can also be used to correctly identify specific road anomalies and obstacles on the road infrastructure, which can be used as landmarks in maps to improve the positions of vehicles (including autonomous vehicles) in the future.

Future developments will extend the scope of the study presented in this paper in various directions. One direction in the data collection phase is to use additional sensors’ inputs (e.g., different placements of the accelerometers/gyroscopes in the vehicle, shock absorber responses of the four absorbers, or the wheels’ speed). Another direction in the data collection phase would be to collect the data with different passengers and drivers in the vehicles. One direction in the processing phase is to investigate the application of additional time-frequency transformations in combination with other deep learning architectures and algorithms. Future developments will also investigate the combination of thresholds or smoothing filters with CNN-SP to enhance the precision and recall results for the classification of the NORM segments. In particular, we will investigate adaptive filters whose definition and parameters are modified depending on the conditions of the road or the vehicle model.

## Figures and Tables

**Table 1:** Order and specifications(brand and model) of the cars used in the data collection.

Car	Manufacturer	Model	Generation	Version
1	Fiat Automobiles (Turin, Italy)	Panda	2nd	Active
2	Fiat Automobiles (Turin, Italy)	Panda	2nd	Active
3	Fiat Automobiles (Turin, Italy)	Panda	2nd	Active
4	Fiat Automobiles (Turin, Italy)	Panda	2nd	Active
5	Fiat Automobiles (Turin, Italy)	Panda	2nd	Active
6	Fiat Automobiles (Turin, Italy)	Panda	2nd	Active
7	Fiat Automobiles (Turin, Italy)	Punto	2nd	3-door
8	Fiat Automobiles (Turin, Italy)	Doblo	1st	Facelift
9	Fiat Automobiles (Turin, Italy)	Tipo	3rd	Hatchback
10	Mitsubishi (Tokyo, Japan)	Colt	6th	CZ3
11	Škoda Auto (Mladá Boleslav, Czech Republic)	Octavia	3rd	Estate
12	Mazda Motor Corp. (Hiroshima, Japan)	Mazda3	4th	Hatchback

**Table 2:** Technical specificationsof the sensor used in the data collection: Xsens with Model Number MTi−100−2A8G4.

**Accelerometer**		
*Parameter*	Measurement unit	Value
Standard full range	m/s${}^{2}$	200
Initial bias error	m/s${}^{2}$	0.05
In-run bias stability	μg	15
Bandwidth (−3 dB)	Hz	375
Non-linearity	%	0.01
**Gyroscope**		
Parameter	Measurement unit	Value
Standard full range	${}^{\circ }$/s	450
Initial bias error	${}^{\circ }$/s	0.2
In-run bias stability	μg	10
Bandwidth (−3 dB)	Hz	415
Non-linearity	%	0.1

**Table 3:** Hyperparameters values and ranges of the adopted machine learning algorithms.

Machine Learning Algorithm	Hyperparameter Name	Optimal Hyperparameter Value	Optimal Hyperparameter Range
CNN	Number of filters (*Nf*)	Nf=30	20, …, 40
CNN	Solver	RMSProp	Choice between RMSProp, stochastic gradient descent and stochastic gradient descent with momentum
CNN	*L2*	0.001	0.0001, …, 0.01
CNN	Batch size	128	64, …, 256
CNN	Number of Convolutional layers (*NC*)	NC=3	2, …, 5
SVM	Kernel	RBF	Choice between linear, RBF, and polynomial (orders 2 and 3) kernels
SVM	RBF scaling factor	$\gamma =2^{5}$	$2^{2}$, …, $2^{10}$
SVM	*C* factor	$C=2^{7}$	$2^{2}$, …, $2^{10}$
KNN	*K* factor	5	1, …, 20
KNN	Distance	Euclidean distance	Choice between Euclidean, Manhattan, Chebyschev, and Mahalanobis distances

**Table 4:** Accuracy obtained for the different representations at 50 Hz. Bold represents the highest value.

Machine Learning Algorithm and Representation	Accuracy
CNN-SP (magnitude) (Hamming window $O_{lap}=66\%,W_{R}=5$)	**0.9720**
CNN with STFT (phase) (Hamming window $O_{lap}=66\%,W_{R}=5$)	0.8047
CWT-CNN (magnitude and Morlet wavelet)	0.9421
CWT-CNN (phase and Morlet wavelet)	0.7812
CNN-1D	0.9354
SVM	0.7422
KNN	0.6728

## Abbreviations

The following abbreviations are used in this manuscript:

1D-T	One-Dimensional Time domain
CNN	Convolutional Neural Network
CNN-1D	Convolutional Neural Network-One-Dimensional
CNN-SP	Convolutional Neural Network-Spectrogram
CWT	Continuous Wavelet Transform
ECOC	Error-Correcting Output Codes
FP	False Positive
FN	False Negative
ENU	East-North-Up
GNSS	Global Navigation Satellite System
IMU	Inertial Measurement Unit
KNN	K Nearest Neighbor
LiDAR	Light Detection And Ranging
RPY	Role-Pitch-Yaw
RBF	Radial Basis Function
RF	Road Feature
SB	Speed Bump
STFT	Short Time Fourier Transform
SVM	Support Vector Machine
TN	True Negative
TP	True Positive
